# Introduction to the *Toxins* Special Issue on Plant Toxins

**DOI:** 10.3390/toxins7114503

**Published:** 2015-11-02

**Authors:** Nilgun E. Tumer

**Affiliations:** Department of Plant Biology and Pathology, School of Environmental and Biological Sciences, Rutgers University, New Brunswick, NJ 08901, USA; E-Mail: tumer@aesop.rutgers.edu; Tel.: +1-848-932-6359

Plants express a variety of toxic proteins which are thought to have a role in defense against pathogens and insects. Plant toxins include ribosome inactivating proteins (RIPs), such as ricin toxin, which possess highly specific rRNA *N*-glycosidase activity and have the potential to be used as biothreat agents. The antiviral activity and cytotoxicity of plant toxins have been exploited for research purposes and they have been used in antiviral and targeted cancer therapy. To date, it is not clear how structural differences between these toxins affect receptor binding, intracellular trafficking, ribosome interactions and toxin activity. It is important to understand how their structure-function relationships affect these processes. This Special Issue covers recent progress in different aspects of plant toxin biology from the basic science to medical applications and highlights important aspects of the field.

RIPs disrupt host translation by a single covalent modification, the depurination of a highly conserved adenine from the α-sarcin/ricin loop (SRL) in the large rRNA, resulting in inhibition of translation. RIPs can be single chain (type 1) such as pokeweed antiviral protein (PAP) from *Phytolacca americana* L. and trichosanthin (TCS) from *Trichosanthes kirilowii* L. or double chain (type 2), such as ricin from the seeds of castor bean, *Ricinus communis* L. and abrin from *Abrus precatorius* L. Type 2 RIPs consist of a cell binding B chain linked to the catalytic A chain by a disulfide bond. There are RIPs that cannot be grouped into the type 1 and type 2 RIPs. The review by Schrot *et al.*, proposes a classification scheme for all known plant RIPs, including those that cannot be classified as type 1 or type 2 RIPs [1].

Ricin is extremely toxic; a single molecule can inactivate 1500 ribosomes per minute. Presently there are no post exposure therapeutics or vaccines available against exposure to ricin. Because of its high potency and the lack of antidotes, ricin has been used as a bioterrorist weapon and remains a threat worldwide. Ricin follows the retrograde pathway from endosomes to the *trans*-Golgi network (TGN) and subsequently enters the endoplasmic reticulum (ER). Accumulated evidence suggests that ricin A chain (RTA) uses components of the ER-associated degradation (ERAD) pathway to reach the cytosol. The details of ricin trafficking [2], host cell signal transduction [3], ribosome binding [4] and the induction of apoptosis by ricin [5] have been recently reviewed. Retrograde trafficking of RTA has been examined using a reconstituted holotoxin composed of C-terminally tagged RTA bearing a motif that can be sulfated in the Golgi stack [6]. The review by Spooner and Lord [7] points out potential caveats that need to be taken into account with sulfated RTA and focuses on the molecular events that have been elucidated using non-tagged ricin and its isolated subunits at the ER-cytosol interface [7]. Evidence is presented that the C-terminus of RTA, which interacts with cellular components within the ER membrane, may be more important than previously recognized [7].

Once RIPs enter the cytosol, ribosomal proteins facilitate their action on ribosomes. Recent studies showed that ricin, Shiga toxin 1 and TCS bind to the ribosomal stalk to access the SRL [4]. The review by Choi *et al*., highlights structural studies which provide insights into how TCS is recruited to the ribosome [8]. Since TCS interacts with the ribosomal stalk, which together with the SRL constitutes the GTPase activation center involved in binding of the elongation factors and activation of GTP hydrolysis [4], the authors propose that TCS may be hijacking the translation factor recruitment function of the ribosomal stalk to reach its target site on the ribosome [8].

Studies of the chronic effects of RIP exposure and its mitigation are important for limiting post-exposure damage and the development of vaccines and therapeutics. Ebulin from *Sambucus ebulus* L. is a type 2 RIP, similar to ricin, but is much less toxic than ricin even though it has the same enzymatic activity on the ribosome. In this Special Issue, Jimenez *et al*., review the functional characteristics of ebulin [9] and the article by Garrosa *et al*., describe the age-dependent toxic effects of ebulin f on the lungs and intestines of mice [10]. The article by Pincus *et al*. [11] describes pathological observations in animals exposed to lethal aerosolized ricin and indicates that respiratory failure, which occurs within 28–48 h after aerosol exposure is the most likely cause of death. Exposure to sublethal doses of aerosolized ricin results in recovery from acute lung disease but causes signs of long-term injury. The authors suggest that humans are likely to survive a ricin attack due to either low dose exposure or early administration of supportive therapy. The information presented in this article is potentially useful to physicians seeking to provide therapy to victims of aerosolized ricin.

Type 1 RIPs, which lack the B chain are much less toxic than type 2 RIPs. There is evidence that some RIPs do not exclusively act on ribosomes and can target different nucleic acid substrates. Most type 1 RIPs have antiviral, antifungal and insecticidal properties, suggesting a role in plant defense against pathogens and insects [12,13]. The mechanism of anti-pathogen activity of RIPs is largely unknown. It has been suggested that they limit pathogen spread from infected cells by eliciting host cell death. However, there is evidence that the antiviral activity of PAP does not depend solely on ribosome inactivation [14,15]. PAP has been shown to inhibit the *in vitro* translation of brome mosaic virus (BMV) and potato virus X (PVX) RNAs by binding to the cap structure and depurinating the viral RNA [15]. Recent results indicate that PAP depurinates the HIV-1, HTLV-1 and BMV RNAs, inhibiting several steps in the viral life cycle [14]. TCS depurinates HIV-1 long terminal repeats, preventing provirus integration [16]. The review by Domashevskiy and Goss [17] focuses on the structure and function of PAP and its antiviral activity and addresses the inhibitory effects of PAP on uncapped viruses that contain a viral genome linked protein (VPg) at their 5′ end [17]. The review by Di and Tumer shows that PAP expression induces a wide range of defense responses in transgenic plants, which may contribute to the broad spectrum disease resistance observed in transgenic plants expressing PAP [18]. Wang *et al*., show that an RIP from maize classified as a type 3 RIP improves cell survival and transiently reduces viral load and in simian-human immunodeficiency virus (SHIV) 89.6 infected macaques [16]. The results of this article suggest the potential of maize RIP as an anti-HIV agent. However, questions remain regarding the specificity of RIPs for viral RNAs.

The mechanism of toxicity of plant toxins is of great interest because they are present in foods [9,19], used in ethnomedicine [20], in cosmetics [21] and have broad range of medicinal applications. Toxicity of RIPs to mammalian cells is multifactorial and molecular details of how they enter cells, traffic through ER targeting pathways, enter the cytosol, depurinate the ribosome, activate ribotoxic stress response and apoptosis are not well understood. Despite extensive research these processes have been difficult to unravel due to extreme toxicity of these proteins. There are no therapeutics or vaccines against RIPs. I hope that this Special Issue will provide readers a better understanding of the mechanism of action of plant toxins, their agricultural and medical applications and will highlight the need for a better basic understanding of the mechanism of their toxicity in order to lead to progress in mitigating their toxic effects.
